# Genome-wide association analysis using multiple Atlantic salmon populations

**DOI:** 10.1186/s12711-025-00959-1

**Published:** 2025-02-27

**Authors:** Afees A. Ajasa, Hans M. Gjøen, Solomon A. Boison, Marie Lillehammer

**Affiliations:** 1 Department of Breeding and Genetics, Nofima (Norwegian Institute of Food, Fisheries and Aquaculture Research), P. O. Box 210, N-1431 Ås, Norway; 2 Department of Animal and Aquacultural Sciences, Norwegian University of Life Sciences, 5003 NMBU, N-1432 Ås, Norway; 3 Mowi Genetics AS, Sandviksboder 77AB, Bergen, Norway

## Abstract

**Background:**

In a previous study, we found low persistence of linkage disequilibrium (LD) phase across breeding populations of Atlantic salmon. Accordingly, we observed no increase in accuracy from combining these populations for genomic prediction. In this study, we aimed to examine if the same were true for detection power in genome-wide association studies (GWAS), in terms of reduction in p-values, and if the precision of mapping quantitative trait loci (QTL) would improve from such analysis. Since individual records may not always be available, e.g. due to proprietorship or confidentiality, we also compared mega-analysis and meta-analysis. Mega-analysis needs access to all individual records, whereas meta-analysis utilizes parameters, such as p-values or allele substitution effects, from multiple studies or populations. Furthermore, different methods for determining the presence or absence of independent or secondary signals, such as conditional association analysis, approximate conditional and joint analysis (COJO), and the clumping approach, were assessed.

**Results:**

Mega-analysis resulted in increased detection power, in terms of reduction in p-values, and increased precision, compared to the within-population GWAS. Only one QTL was detected using conditional association analysis, both within populations and in mega-analysis, while the number of QTL detected with COJO and the clumping approach ranged from 1 to 19. The allele substitution effect and -log_10_p-values obtained from mega-analysis were highly correlated with the corresponding values from various meta-analysis methods. Compared to mega-analysis, a higher detection power and reduced precision were obtained with the meta-analysis methods.

**Conclusions:**

Our results show that combining multiple datasets or populations in a mega-analysis can increase detection power and mapping precision. With meta-analysis, a higher detection power was obtained compared to mega-analysis. However, care must be taken in the interpretation of the meta-analysis results from multiple populations because their test statistics might be inflated due to population structure or cryptic relatedness.

**Supplementary Information:**

The online version contains supplementary material available at 10.1186/s12711-025-00959-1.

## Background

Genome-wide association studies (GWAS) can help provide insight into the genetic architecture of complex traits. The success of GWAS depends on several factors such allele frequency, allele substitution effect size, sample size, and degree of complexity of the trait [1]. Of these factors, it is only sample size that is within the control of the investigator. GWAS in aquaculture populations have so far often been based on small sample sizes, usually less than or around 1000 [2–4], probably due to the fairly recent adoption of genomic technologies for aquaculture species compared to other livestock species, and the cost associated with genotyping and phenotyping. In addition, unlike livestock populations, data from overlapping generations are often not combined. A plausible approach to increase sample size, and consequently the power to detect quantitative trait loci (QTL), is to combine data from multiple studies or populations, which can be done by pooling individual genotypes and phenotypes from multiple studies or populations together (mega-analysis). Alternatively, if there are restrictions on sharing individual data, due to proprietorship or confidentiality, summary statistics from different GWAS can be aggregated in a meta-analysis, an example is the study of stature in cattle [5], and other studies on humans. Indeed, studies have shown that these two methods give similar results [6, 7]. Different meta-analysis methods are available, differing in the parameters used in the analysis and the assumption of allele substitution effect across studies or populations [8]. Furthermore, meta-analysis also facilitates the study of the consistency of allele substitution effect across studies or populations [8, 9].

An important factor that can inhibit the expected increase in detection power, which should be normally obtained from an increased sample size when combining multiple studies or populations, is the persistency of linkage disequilibrium (LD) phase across studies or populations, particularly when medium or low-density single nucleotide polymorphisms (SNPs) data is used, as the causal variants will likely not be genotyped. When LD phase is poorly conserved across populations, associations between markers and QTL may be lost or cancelled out in a mega or meta-analysis. However, this can help refine QTL's position [10].

A common practice in GWAS is to select the lead SNP as the putative QTL and to regard other significant SNPs within the same region as being in LD with the QTL. However, it is plausible that there are multiple QTL, often referred to as independent or secondary signals, in the same genomic region. The gold standard for determining the presence or absence of secondary signals is arguably conditional association analysis, where the top SNP, i.e. lead-variant, is included as a fixed effect in the model, until no further significant association is found. However, this method cannot be utilized with summary statistics usually obtained from meta-analysis. Nonetheless, Yang et al. [11] presented an approximate conditional and joint analysis (COJO), that can be utilized with summary statistics. Another method for detecting secondary signal(s) that also works with summary statistics is the clumping approach, where secondary signals are identified based on LD and physical distance of significant SNPs.

To investigate the potential benefits of using the approaches described above, this study aims to (i) determine if increased detection power of QTL, in terms of reduction in p-values, and refined QTL position(s) can be derived from combining populations with low persistence of LD phase in a GWAS (mega-analysis), (ii) identify secondary signals, (iii) identify which meta-analysis method approximate mega-analysis the best.

## Methods

### Phenotypes

**Fig. 1:**
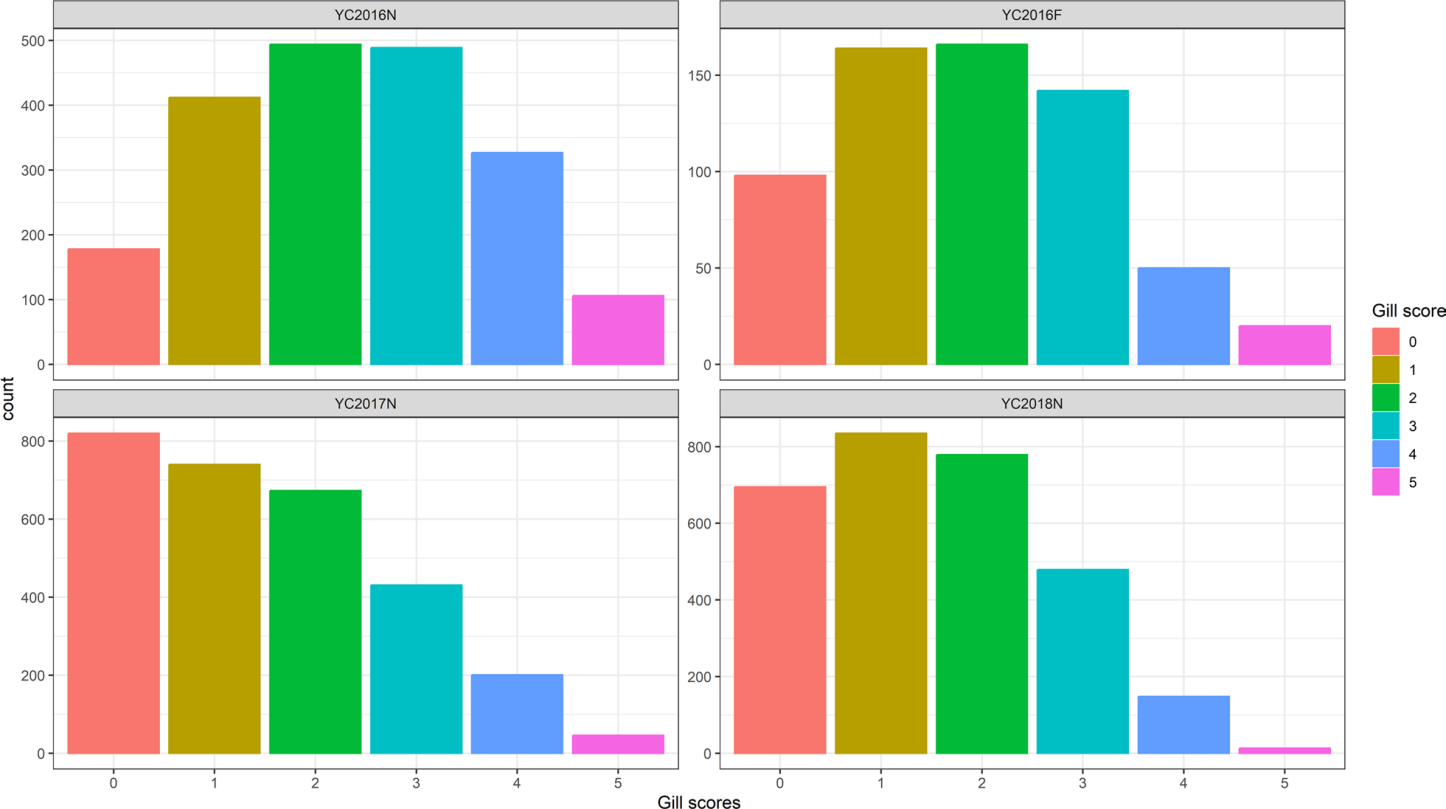
A bar chart of gill scores for the various populations

The dataset used in this study has been described previously in Ajasa et al. [12, 13]. Briefly, the phenotype is a categorical gill score (0–5; 0 indicating no infection, 5 indicating severe infection) of four Atlantic salmon populations from three year-classes (YC, denoting the year they were put to sea) recorded during outbreaks of amoebic gill disease (AGD), a parasitic disease of significant concern to the salmon industry worldwide. The populations used in this study are YC2016N, YC2016F, YC2017N, and YC2018N. These populations were all from the fully integrated salmon company Mowi and comprised mainly of the Norwegian nucleus populations, indicated by the letter N appended to YC’s name. However, one population in the year class 2016 originated from Mowi’s Irish (Fanad) populations, as indicated by the letter F appended to its name. A descriptive statistics of gill scores for each population are shown in Table 1, and a bar chart of the gill scores categories for each population is shown in Fig. 1.

**Table 1:** Descriptive statistics of gill scores for the various populations

	YC2016N	YC2016F	YC2017N	YC2018N
Number of records	2006	640	2911	2949
Mean (SD)	2.35 (1.35)	1.91(1.30)	1.52 (1.31)	1.52 (1.18)

SD, standard deviation

### Genotypes

**Fig. 2:**
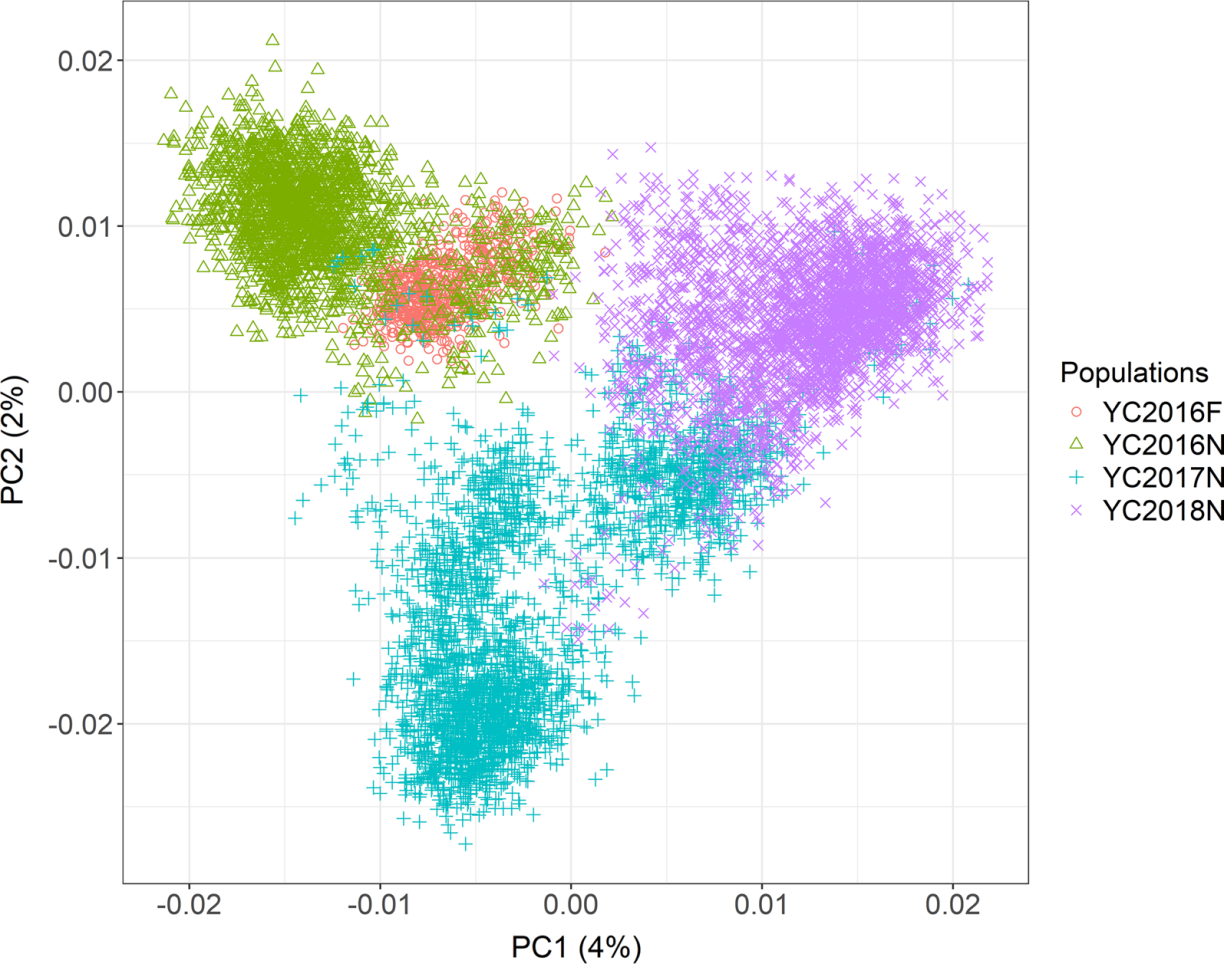
Principal component analysis of all populations based on genomic relationship matrix using GCTA [15]

All populations were genotyped with a 55 k SNP chip developed by Nofima in collaboration with SalmoBreed and Mowi. Quality control was performed jointly, as described previously in Ajasa et al. [12]. Briefly, it involves removing individuals or markers with a call rate < 95%, minor allele frequency < 1%, and Hardy Weinberg p-value (Fisher’s exact test) < 10*e*−25. Finally, only samples with heterozygosity frequency between 0.25 and 0.45 were retained, so as to limit the impact of poor-quality samples [14]. After quality control and imputation of sporadic missing genotypes, 50,456 SNPs remained. Principal component analysis (PCA) of all populations based on the genomic relationship matrix is shown in Fig. 2. The average genetic relationship and genetic distance between populations are shown in Additional file 1: Table S1a and b, respectively.

### Genome-wide association study

#### Within population

For each population, the following model was used for analysis

$$\mathbf{y}=1\upmu +\mathbf{x}\text{b}+\mathbf{Z}\mathbf{u}+\mathbf{e},$$

where **y** is the vector of gill scores, $\upmu$ is the overall mean, **x** is the vector of SNP genotypes (coded 0|AA, 1|AG, 2|GG), b is the allele substitution effect, **Z** is an incidence matrix relating the phenotype to the polygenic effects **u**, and **e** is a vector of the residual effects. **u** ~ N (**0**, **G** ${\sigma }_{\text{u}}^{2}$), **e** ~ N (**0**, **I** ${\sigma }_{\text{e}}^{2}$), where **I** is an identity matrix, ${\sigma }_{\text{e}}^{2}$ is the residual variance, **G** is the genomic relationship matrix (GRM), and ${\sigma }_{\text{u}}^{2}$ is the additive genomic variance. Unless stated otherwise, all the GWAS was performed with GCTA (–mlma option) [15]. Genomic inflation factor (λ) was estimated from the median chi-square statistics of the p-values divided by the expected median chi-square (0.455) under the null distribution. λ values > 1.1 are usually considered as evidence of confounding [16], which may be due to population structure or other non-random artifacts [17, 18]. A false discovery rate (FDR) of < 0.05 was used to correct for multiple testing using the Benjamini-Hochberg (BH) procedure [19], which involves ranking markers based on their p-values in ascending order (*P*_*i,*_…,*P*_*m*_), then the highest rank where ${P}_{i}<\alpha \frac{k}{m}$ is found, and all markers with ranks lower or equal to this are declared significant. $\alpha$ is the set significance threshold, *k* is the rank of marker *i*, and *m* is the number of markers. This was implemented using the *p.adjust* function in R [20]. The output from this function is a BH-adjusted p-value which is not suitable for the various secondary signals determination methods studied. Hence, the corresponding p-value threshold at FDR < 0.05 was derived following Bolormaa et al. [21].

### Multi-population

#### Mega-analysis

Phenotype and genotype data for all populations were combined for GWAS with the same model as described above but including a fixed effect of population.

#### Meta-analysis

Meta-analysis approaches differ mainly on the parameters used, i.e. p-values or allele substitution effects [8]. The most widely used meta-analysis method based on p-value is the Z-score method [22], whereas the allele substitution effect meta-analysis methods are grouped into two: fixed effects and random effects, which differ in their assumptions of allele substitution effects. While the fixed effects model assumes that the allele substitution effects are the same across studies or populations, the random effects model allows the allele substitution effects to differ across populations [8]. Inverse variance weighted (IVW) is the most widely used type of fixed effects meta-analysis. The meta-analysis methods used in this study include Z-score, IVW, and random effects meta-analyses.

#### Z-score

Z-score meta-analysis uses p-values from each study or population to estimate the Z-score, and each study is weighted by its sample size. The drawback of this approach is that it does not provide an estimate of the allele substitution effect. The Z-score meta-analysis was performed using a custom-made R function zscore_meta.R [23]. The Z-score (*Z*) and overall *P* are computed as follows:

$$Z=\frac{{\sum }_{j=1}^{N}{Z}_{ij}{w}_{ij}}{\sqrt{{\sum }_{j=1}^{N}{{w}_{ij}}^{2}}}, P=2\varnothing (\left|-Z\right|),$$

where *w*_*ij*_ is the square root of the sample size of the *i*^*th*^ SNP of the *j*^*th*^ study or population, *N* is the number of study or population, and *Z*_*ij*_ = ${\boldsymbol{\varnothing }}^{-1}\left(1-\boldsymbol{ }\frac{{P}_{{\varvec{i}}{\varvec{j}}}}{2}\right)\times(\textit{sign or direction of allele substitution effect})$**,** where $\varnothing$ is the cumulative distribution function, *Z*_*ij*_ and *p*_*ij*_ are the z-score and p-value of the *i*^*th*^ SNP of the *j*^*th*^ study or population, respectively.

#### Inverse variance weighted (IVW)

IVW weighs each study or population by the inverse of the variance of the allele substitution effects. The variance of allele substitution effects is a function of heritability, allele frequency and sample size [24]. This model was implemented with the GWAMA software [25] based on:

$${\beta }_{j}=\frac{{\sum }_{j=1}^{N}{\beta }_{ij}{w}_{ij}}{{\sum }_{j=1}^{N}{w}_{ij}}, {w}_{ij}={V}_{ij}^{-1},{V}_{ij}\hspace{0.17em}=\hspace{0.17em}var({\upbeta}_{ij}),$$

where ${\beta }_{ij}$ is allele substitution effect for SNP *i* and study or population *j*, ${w}_{ij}$ is the weight for SNP *i* and study or population *j*, *N* is the number of studies or populations, and ${\beta }_{j}$ is the combined allele substitution effect across studies or populations. GWAMA [25] also provides two metrics for heterogeneity: Cochran’s heterogeneity (Q) and heterogeneity index (I^2^) statistics for each SNP. Q and I^2^ are widely used metrics for measuring the presence and amount of heterogeneity in GWAS [8]. Cochran’s Q measures the amount of between-study heterogeneity of allele substitution effects, and it follows a chi-square distribution with *N-1* degrees of freedom at the significance level of 0.10. However, it has less statistical power when the combined studies are small. I^2^ measures the proportion of heterogeneity of allele substitution effects that is not due to chance and it is not affected by the number of studies [8]. Values of I^2^ < 25 indicate no heterogeneity, 25–50: moderate heterogeneity, 50–75: high heterogeneity, and > 75: very high heterogeneity [26].

$$\text{Q }= {\sum }_{j=1}^{N}{w}_{ij}({\beta }_{ij}-{\beta }_{j}{)}^{2},  {I}^{2}=\frac{\text{Q}-\left(\text{N}-1\right)}{Q} \times 100.$$

The variance of the combined allele substitution effect ${\beta }_{j}$ is given by V_*j*_ $={\left[{\sum }_{j=1}^{N}{w}_{ij}\right]}^{-1}$. The test statistics ${\chi }_{j}$^2^ = $\frac{{{\beta }_{j}}^{2}}{{V}_{j}}$ is from a chi-square distribution with one degree of freedom [25].

#### Random effects

The random effects model incorporates between study or population variance (${\tau }^{2}$) [27] in the estimation of the weights for each study. Due to its random effect nature, its estimates can be generalized across different studies or populations [8]. When ${\tau }^{2}$ = 0, the estimates from random and IVW models are the same. In the presence of heterogeneity, the conventional random effect model has low power to detect heterogeneous genetic effects [9, 28], hence Han and Eskin [28] developed a new random effect model that is better suited for such cases. This model was implemented here using the METASOFT software [28]. Its test statistics (*S*_rand_) is decomposable into two parts comprising the test statistics (*S*_FE_) for the fixed effect model and the test statistics (*S*_Het_) when there is heterogeneity, i.e. ${\tau }^{2}\ne$ 0,

$$S_{{{\text{rand}}}} \, = \,S_{{{\text{FE}}}} \, + \,S_{{{\text{Het}},}}$$

$$S\text{rand }=\left\{\sum_{j=1}^{N}\frac{{\beta }_{ij}^{2}}{{V}_{ij}}-\sum_{j=1}^{N}\frac{{({\beta }_{ij}- {\beta }_{j})}^{2}}{{V}_{ij}} \right\}+\left\{\sum_{j=1}^{N}log\left(\frac{{V}_{ij}}{{V}_{ij}+ {\tau }^{2}}\right)+ \sum_{j=1}^{N}\frac{{({\beta }_{ij}- {\beta }_{j})}^{2}}{{V}_{ij}}- \sum_{j=1}^{N}\frac{{({\beta }_{ij}- {\beta }_{j}^{*})}^{2}}{{V}_{ij}+ {\tau }^{2}}\right\},$$

$${\beta }_{j}^{*}=\frac{{\sum }_{j=1}^{N}{\beta }_{ij}{w}_{ij}^{*}}{{\sum }_{j=1}^{N}{w}_{ij}^{*}},w_{ij}^{*} = {[{\tau }^{2}+ {V}_{ij}]}^{-1},$$

$${\tau }^{2}=\frac{\text{Q}-(\text{N}-1)}{{\sum }_{j=1}^{N}{w}_{ij} - \frac{{{\sum }_{j=1}^{N}{w}_{ij}}^{2 }}{{\sum }_{j=1}^{N}{w}_{ij}}}$$ [29].

All parameters are the same as previously defined. *S*_FE_ is equivalent to the test statistics of the IVW model described earlier [28].

### Independent or secondary signal(s) identification

The three methods described below are to identify independent or secondary signal(s). In other words, to determine if multiple QTL are within the same region.

#### Conditional association analysis

In this analysis, we fitted the lead significant variant as an additional covariate in the GWAS model described above for within-population and mega-GWAS. If no additional variant is identified after this step, the first and last SNP position of the significant SNPs in the previous step is used to define the QTL’s interval or boundary. Otherwise, the newly identified lead significant SNP is fitted as an additional covariate in the model, and the QTL interval is calculated by subtracting the QTL boundary of the first step from that of the subsequent step. These steps are repeated until no significant variant is found [30]. Since conditional association analysis is the gold standard [31, 32], it would serve as a benchmark against which other methods are evaluated.

#### COJO

The COJO method [11] starts with the most significant (lead) SNP based on a predefined significance threshold, and then new p-values are obtained by conditioning the effect of other SNPs on this SNP. The SNP with the lowest conditional p-value, below the set significance threshold, is then selected, provided that it is uncorrelated with the lead SNP (squared correlation of < 0.9 by default, which can be adjusted with the *–cojo-collinear* function in GCTA [15]). Otherwise, it is dropped by setting the conditional p-value to 1. The selected SNPs are then fitted jointly in the model, dropping the largest non-significant SNP based on the set threshold. The above steps are then repeated until no SNP can be added or removed. COJO for both within and multi-population GWAS results was performed in GCTA [15] with the function *cojo-slct*. The reference population used for COJO is the raw genotype data of the analyzed population for the within-population GWAS, while for the mega and meta-analysis, all the raw genotype data were used. The COJO in GCTA [15] requires allele substitution effect (*b*) and standard error (*SE*) estimates, which are not available for the Z-score meta-analysis method. Hence, *b* and SE were estimated using a formula presented by Zhu et al. [33] below:

$$b=\frac{z}{\sqrt{2p(1-p)(n+{z}^{2})}}, SE=\frac{1}{\sqrt{2p(1-p)(n+{z}^{2})}},$$

where *p* is the minor allele frequency of the SNP, *n* is the sample size, and *z* is the z-score. The default setting in GCTA for COJO also assumes that SNPs are in linkage equilibrium (LE) at 10 MB, which is not true for aquaculture breeding populations, due to extensive long-range LD [12, 34]. Yengo et al. [35], showed that estimates from COJO can be biased when there is long-range LD in the studied population and recommended using a strict collinearity threshold in such instances. Similarly, Veerkamp et al. [36], noted that the family structure in livestock populations (small effective population size) causes inflated and overestimated conditional summary statistics from COJO. Hence, to avoid these issues, we assumed that markers 100 MB away from each other are in LE, using the *–cojo-wind* function, as in van den Berg et al. [37], and used a strict collinearity threshold of 0.05.

#### Clumping

As stated earlier, for this approach, independent or secondary signals are defined based on LD and distance between significant markers. The *clumping* approach was implemented in Plink [38] with the following parameters; *–clump-p1* < *p-value threshold* > *, –clump-r2 0.05, –clump-kb 5000.* Clumps are formed around the lead variants based on p-value threshold, LD (measured by r^2^), and physical distance. The LD and physical distance values were set arbitrarily. *–clump-p1* denotes the maximum p-value for variants in a clump, *clump-r2* sets the minimum *r*^*2*^ between the lead variants and other variants in a clump, *–clump-kb* sets the maximum distance between lead variants and other variants in a clump. The algorithm starts the first clump with the most significant variant, other variants that meet the criteria set above are also included in the first clump. The next clump is formed with the most significant variant that was not included in the first clump/group and that meets the set clumping threshold. The algorithm iterates until no significant variants remain.

## Results

**Fig. 3:**
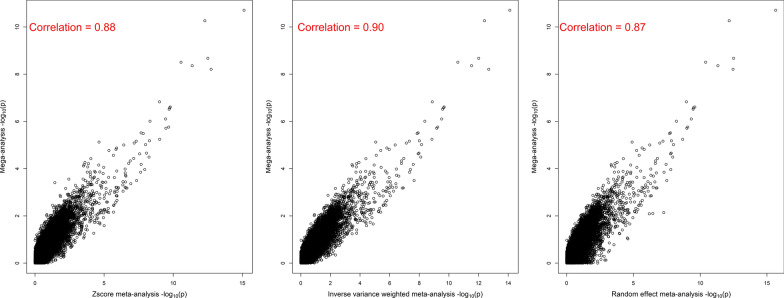
A plot of raw −log_10_p-values obtained from mega and meta-GWAS of susceptibility to amoebic gill disease (AGD)

**Fig. 4:**
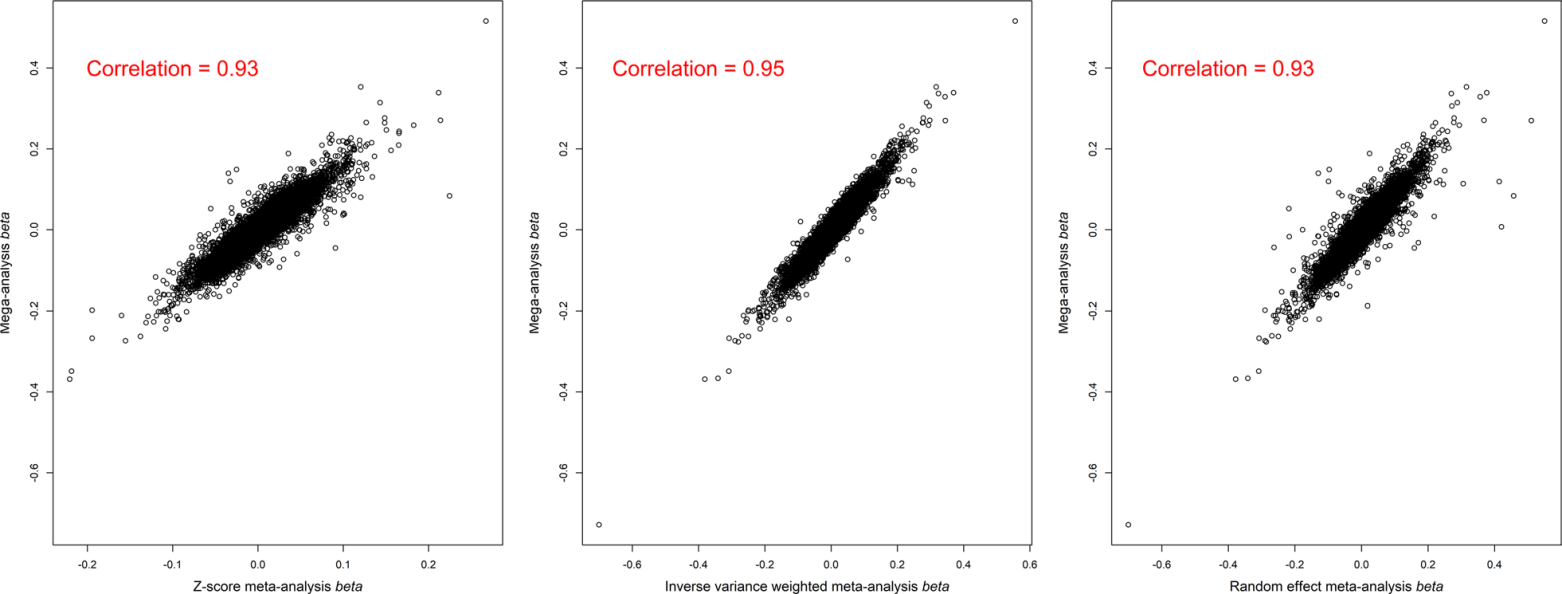
A plot of allele substitution effect (beta) obtained from mega and meta-GWAS of susceptibility to amoebic gill disease (AGD)

Plots of the -log_10_p-values and allele substitution effects from the mega-analysis and the various meta-analysis models are shown in Figs. 3 and 4, respectively. The -log_10_p-values from the various meta-analysis models were highly correlated with the mega-analysis results, with correlations ranging from 0.87 to 0.90, while the correlations of allele substitution effects estimated with each of the different methods were: 0.93 between mega and Z-score meta-analysis, 0.95 between mega and IVW meta-analysis, and 0.93 between mega and random effect meta-analysis.

**Fig. 5:**
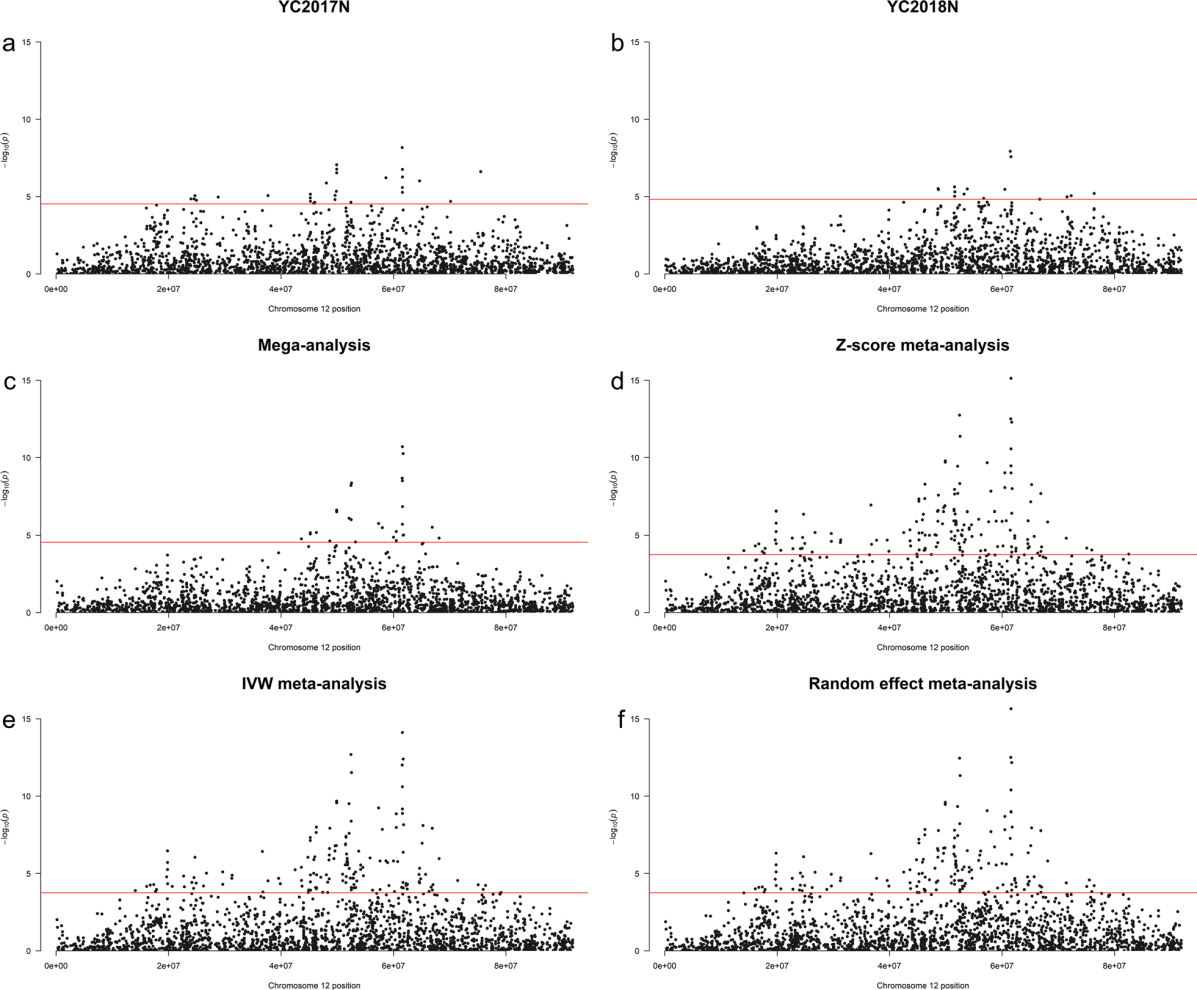
− log_10_p-values of GWAS for chromosome 12 for YC2017N (**a**), YC2018N (**b**), Mega-analysis (**c**), Z-score meta-analysis (**d**), inverse variance weighted (IVW) meta-analysis (**e**), and random effect meta-analysis (**f**) at FDR < 0.05 shown with red line

Table 2 gives the summary of the within-population GWAS results for populations with significant QTL regions. YC2016N and YC2016F had no significant markers. Based on the FDR of 0.05, 5% of the significant SNPs are expected to be false positives. Hence, we focused on the region with more than two significant SNPs as well detected both within populations and with mega-GWAS. The highest number of putative QTL(s) were found using the clumping approach while the lowest number was found from the conditional association analysis. The within population λ estimate was ~ 1, providing no evidence of inflated test statistics*.* The summary statistics for significant SNPs within populations are shown in Additional file 1: Table S2, and the Manhattan plot for each population is shown in Additional file 2: Figures S1–S4. The -log_10_p-values for chromosome 12 for within and multi-population GWAS are shown in Fig. 5.

**Table 2:** Summary of the within-population GWAS

Population	λ	nSNPs	QTL interval or boundary (bp)	Highest -log_10_p-value	nQTL		
					CA	COJO	Clumping
YC2017N	0.96	28	51,560,918	8.18	1	1	7
YC2018N	0.96	15	27,753,777	7.93	1	1	5

nSNPs, number of significant SNPs; nQTL, number of putative QTL or independent signals identified; CA, conditional association analysis

The summary of the multi-population GWAS results is presented in Table 3. As shown by the -log_10_p-values, the highest detection power and number of significant SNPs were derived via meta-analysis. The highest -log_10_p-value was derived by using the random effect meta-analysis. Again, the highest number of putative QTL was detected from the clumping approach. Manhattan plots for multi-population GWAS are shown in Additional file 3: Figures S5-S8. The summary statistics for significant SNPs in the mega- and meta-GWAS are shown in Additional file 1: Table S3. When the direction of allele substitution effect was not consistent across populations, the YC2016F was in most cases the outlier, probably due to its sample size (sample size is inversely proportional to standard error).

**Table 3:** Summary of the multi-population GWAS and meta-analysis

Analysis type	λ	nSNPs	QTL interval or boundary (bp)	Highest -log_10_ p-value	nQTL		
					CA	COJO	Clumping
Mega-GWAS	0.96	28	24,494,064	10.71	1	2	5
Z-score	1.12	152	68,455,638^1^	15.12	–	4	18
IVW	1.12	154	65,065,463^1^	14.12	–	4	19
Random effect	1.68^2^	159	60,273,936^1^	15.65	–	3	19

nSNPs, number of significant SNPs; nQTL, number of putative QTL or independent signals identified; CA, conditional association analysis

^1^QTL interval was defined based on the number of QTL identified from the mega-GWAS conditional association analysis being the gold standard

^2^Estimated based on the sum of λ from the *S*_*FE*_ and *S*_*Het,*_ see Methods

The majority of the significant SNPs had consistent allele substitution effect estimates across studies or populations. Specifically, the I^2^ metrics of the variants passing the significance threshold with the IVW method, reveal that about 78 (51%) of the variants had homogenous effects across populations. Of the 76 (49%) variants showing heterogeneity, 32 (42%) had moderate heterogeneity, 35 (46%) had high heterogeneity, and 9 (12%) had very high heterogeneity.

## Discussion

This study aimed to determine if an increase in detection power, in terms of reduced p-values, and increased precision can be achieved from GWAS combining different populations of Atlantic salmon gill scored during outbreaks of AGD.

Complex traits are controlled by a large number of genes [39], with each having a small effect, which requires a large number of samples to detect these genes. A larger sample size can be obtained by combining multiple studies or populations using mega or meta-analysis. However, this does not necessarily translate to increased power, as LD phase may not be conserved across studies or populations, among other reasons. For markers that are far from the QTL, evidence of association may be weakened or leveled out by combining multiple studies or populations, since LD phase is poorly conserved at long distances [40], whereas markers close to a QTL may gain evidence of association thereby increasing mapping resolution. Based on the conditional analysis result, that only one QTL was detected within and across populations, our results suggest that combining multiple populations increased the power of detecting QTL, in terms of reduction in p values, and increased mapping precision, with QTL mapped to shorter intervals in mega-analysis, in contrast to the within-population GWAS where QTL intervals were longer. Similar observations were made by [41, 42], who found that combining different breeds increased power and precision of mapping, provided that QTL is segregating across breeds. Given the close genetic similarity between our study populations (see Additional file 1: Table S1), it would have been expected that combining these populations would not reduce the QTL intervals, as observed by van den Berg et al. [10] when several Holstein populations were combined in a mega-analysis. However, as we have reported previously [13], LD phase is poorly conserved across our study populations due to admixture, hence, the reduced QTL interval from combining our study populations was not surprising. In contrast to van den Berg et al. [10], we did not observe a reduction in the number of variants associated with the QTL in the mega-analysis.

There was a great disparity in the number of QTL detected among the conditional association analysis, COJO, and the clumping approach, particularly for the multi-population GWAS. The relatively higher number of QTL detected by the COJO, in contrast to conditional analysis in the mega-GWAS, could be due to the rather lenient significance threshold (FDR < 0.05) used in the analysis; Jian Yang recommends that a stringent significance threshold should be used when running COJO to avoid false positives (personal communication). However, using a stringent significance threshold could result in some signals being undetected, particularly when the power of detecting secondary signals is low, as shown in a simulation study in cattle, by van den Berg and MacLeod [42], where the number of QTL detected by COJO was smaller than that simulated. Nonetheless, it is important to emphasize that the achieved modest performance of COJO was obtained using custom parameters, taking into consideration the LD pattern in our population, whereas studies [43, 44] that have reported a high number of QTL using COJO in GCTA [15] have used default parameters. The results from the clumping approach are comparable to those usually obtained based on arbitrarily defined thresholds used in livestock GWAS to identify the number of independent QTL [10, 45]. Comparing the number of QTL identified from the clumping approach with that from the conditional analysis shows that the former to a larger extent inflates the number of QTL. Also, given that the detected QTL signal disappeared after conditional analysis, indicates that the QTL region spans all the regions showing signal (equivalent to the defined boundaries of QTL) before the conditional analysis. Hence, the 5 MB used to define the QTL region is too small.

The allele substitution effects and -log_10_p-values of the meta-analysis methods were highly correlated with mega-analysis corresponding values. Although this result attests to the equivalence of meta-analysis and mega-analysis [6], there was a marked difference between the number of significant SNPs detected by meta-analysis and mega-analysis. With meta-analysis, we found more detection power, in terms of lower p-values, and a higher number of significant SNPs compared to mega-analysis, which is contrary to theoretical and empirical results [6, 46]. Additionally, contrary to expectations of improved QTL precision when multiple populations are combined for GWAS [10, 32], which was demonstrated with the mega-analysis result, a wider QTL region was detected with meta-analysis compared to the within-population GWAS. A likely explanation of these findings is confounding, which could be due to population structure or cryptic relatedness [47]. Based on the history of mixing between our populations [12], cryptic relatedness exists among individuals across the study populations, as also illustrated in Fig. 2. Devlin and Roeder [48] noted that when there is population stratification, which can arise due to population structure or cryptic relatedness, the assumption of independence of observations in the linear model is violated, resulting in the inflation of test statistics i.e. p-values become smaller than they should. Similarly, Setakis et al. [49] found that cryptic relatedness can reduce p-values by a factor of 4. Furthermore, Helgason et al. [50] found significant inflation of test statistics in populations with a genetic distance of less than 0.01. A mere comparison of the p-values obtained from mega-analysis and meta-analysis (see Additional file 1: Table S3) shows that the p-values from the latter are lower. More importantly, the λ estimate from meta-analysis indicates that the test statistics are inflated. However, the λ estimate from mega-analysis did not indicate inflated test statistics due to population stratification or structure, because population structure was handled by the GRM in the statistical model used [18]. Several studies [47, 51] have noted that the linear mixed model can handle confounding bias caused by population structure and/or cryptic relatedness. In contrast, with meta-analysis, cryptic relatedness or population structure present across studies or populations are not accounted for. Several authors [31, 52–54] have thus seen this as a major challenge with meta-analysis. Therefore, the higher number of significant SNPs and the resulting higher number of QTL detected from meta-analysis compared to mega-analysis should be interpreted with caution; this is particularly important for aquaculture meta-GWAS where some form of relationship often exists among breeding populations.

In a simulation study of unrelated individuals with no population structure or cryptic relatedness, Yang et al. [55] showed that under polygenic architecture, inflation of test statistics can be expected if the study has sufficient power. Some authors have suggested that due to the small effective population size (long-range LD) in livestock populations, an inflated test statistic is expected, leading them to arbitrarily raise the threshold for a significant λ estimate. However, this argument is defective, as LD is not the sole factor influencing the power of QTL detection. And given that most livestock GWAS lack power to detect all or most segregating QTL [24, 39], there is little justification to expect that inflated test statistics are primarily due to polygenicity. Moreover, the high degree of relatedness in livestock populations, which can cause spurious associations, increases the risk of inflated test statistics [56, 57]. Hence, raising the threshold for declaring inflated test statistics in livestock is not justified.

A widely used method for controlling inflation in test statistics in meta-analysis is genomic control (GC) [48], where the test statistics are divided by the λ estimate. However, some authors [18, 47, 58] have pointed out that although GC can help to restore an appropriate null distribution i.e. make $\uplambda \le$ 1, it may be ineffective in controlling the effect of population stratification. Similarly, in a simulation study, Shmulewitz et al. [59] showed that GC can reduce the power of detecting QTL and may not eliminate spurious associations. A more efficient method for controlling inflation of test statistics in meta-analysis is the linkage disequilibrium score regression (LDSC) method [60], which can also partition inflation due to polygenicity and population structure. However, this method has only been developed for application to human GWAS, and there is thus a need for the development of a similar method for livestock and aquaculture populations.

Based on the I^2^ metrics, the most significant SNP detected exhibited very high heterogeneity, indicating that the same causal variants are probably not segregating in the different populations. Other factors that may influence the heterogeneity of effects across populations include winner’s curse, gene by gene interaction (non-additive gene action), gene-by-environment interaction, differential linkage disequilibrium between markers and causal variants across populations, or poor conservation of LD phase [61]. Based on the origin and history of our study populations [12], differential LD patterns between markers and QTL, and poor conservation of LD phase are the most likely sources of heterogeneity between our populations. It is, however, important to note that I^2^ metrics usually have large confidence intervals when the number of studies is small [9], as is the case here; a high I^2^ is thus not a definite confirmation of heterogeneity, nor is a low I^2^ a guarantee of homogeneity.

## Conclusions

Our results demonstrate that combining multiple Atlantic salmon populations in a genome-wide association study (GWAS) via mega-analysis can increase detection power. Additionally, we found that meta-analysis provides a reasonable approximation of mega-analysis. However, a higher detection power observed in meta-analysis compared to mega-analysis should call for caution, as this may be an indication of confounding due to (un)known similarities among populations or studies. Therefore, there is a need to develop models that can control or mitigate confounding in GWAS meta-analysis. Although the results of inflated test statistics using meta-analyses were based on medium-density SNP data, we do not expect a different trend with high-density SNP or sequence data. Nevertheless, this warrants further investigation. In addition, we do not expect this observation to be trait or species-specific given similar results have been observed in humans [54], and more recently in cattle [62].

## Supplementary Information

**Additional file 1: Table S1a.** Average genetic relationship between populations. The GRM was constructed using Van Raden’s method 1. **b** Mean genetic distance between populations. **Table S2.** Within population summary statistics for significant variants. **Table S3.** Summary statistics of significant variants from mega and meta-GWAS.

**Additional file 2: Figure S1.** Manhattan plot of summary statistics derived from genome-wide association analysis for YC2016N. **Figure S2.** Manhattan plot of summary statistics derived from genome-wide association analysis for YC2016F. **Figure S3.** Manhattan plot of summary statistics derived from genome-wide association analysis for YC2017. Significant SNPs at FDR < 0.05 are shown in green. **Figure S4.** Manhattan plot of summary statistics derived from genome-wide association analysis for YC2018. Significant SNPs at FDR < 0.05 are shown in green.

**Additional file 3: Figure S5.** Manhattan plot of summary statistics derived from mega genome-wide association analysis. **Figure S6.** Manhattan plot of summary statistics derived from Zscore meta-genome-wide association analysis. **Figure S7.** Manhattan plot of summary statistics derived from inverse invariance weighted meta-genome-wide association analysis. **Figure S8.** Manhattan plot of summary statistics derived from random effect meta-genome-wide association analysis.

## Data Availability

The data utilized in this study were provided by Mowi Genetics AS and are not publicly accessible.
