# Supplementary material for: Genome-wide association analysis using multiple Atlantic salmon populations
Source: Genet Sel Evol. 2025 Feb 27;57:9. doi: 10.1186/s12711-025-00959-1 (PMC11869457; doi:10.1186/s12711-025-00959-1)
Supplement: Supplementary file 2 — Additional file 2: Figure S1. Manhattan plot of summary statistics derived from genome-wide association analysis for YC2016N. Figure S2. Manhattan plot of summary statistics derived from genome-wide association analysis for YC2016F. Figure S3. Manhattan plot of summary statistics derived from genome-wide association analysis for YC2017. Significant SNPs at FDR < 0.05 are shown in green. Figure S4. Manhattan plot of summary statistics derived from genome-wide association analysis for YC2018. Significant SNPs at FDR < 0.05 are shown in green. [file 12711_2025_959_MOESM2_ESM.docx]

**Genome-wide association analysis using multiple Atlantic salmon populations**

Afees A. Ajasa^1,2^, Hans M. Gjøen^2^, Solomon A. Boison^3^ and Marie Lillehammer^1^

^1^Nofima (Norwegian institute of Food, Fisheries and Aquaculture research), PO Box 210, N-1431 Ås, Norway

^2^Department of Animal and Aquacultural Sciences, Norwegian University of Life Sciences, 5003 NMBU, N-1432 Ås, Norway

^3^Mowi Genetics AS, Sandviksboder 77AB, Bergen, Norway


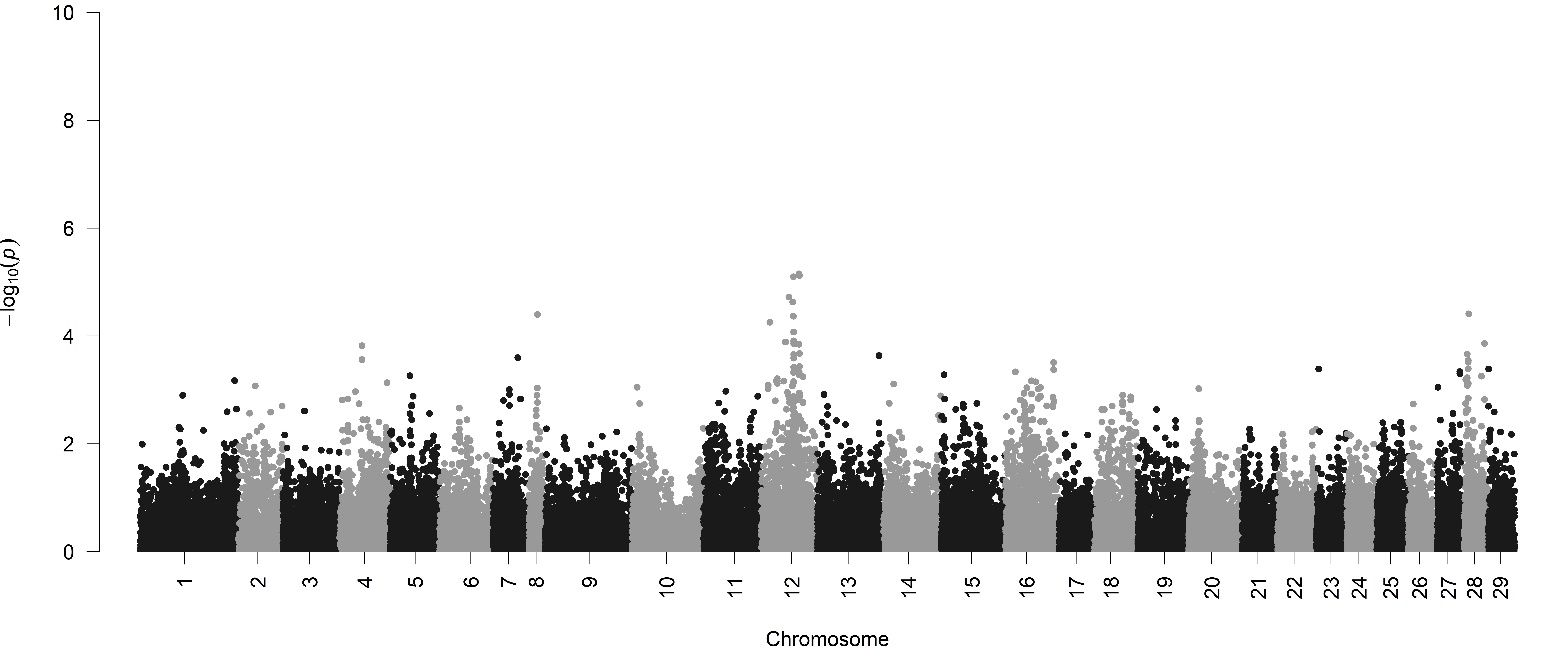


Figure S1: Manhattan plot of summary statistics derived from genome-wide association analysis for YC2016N


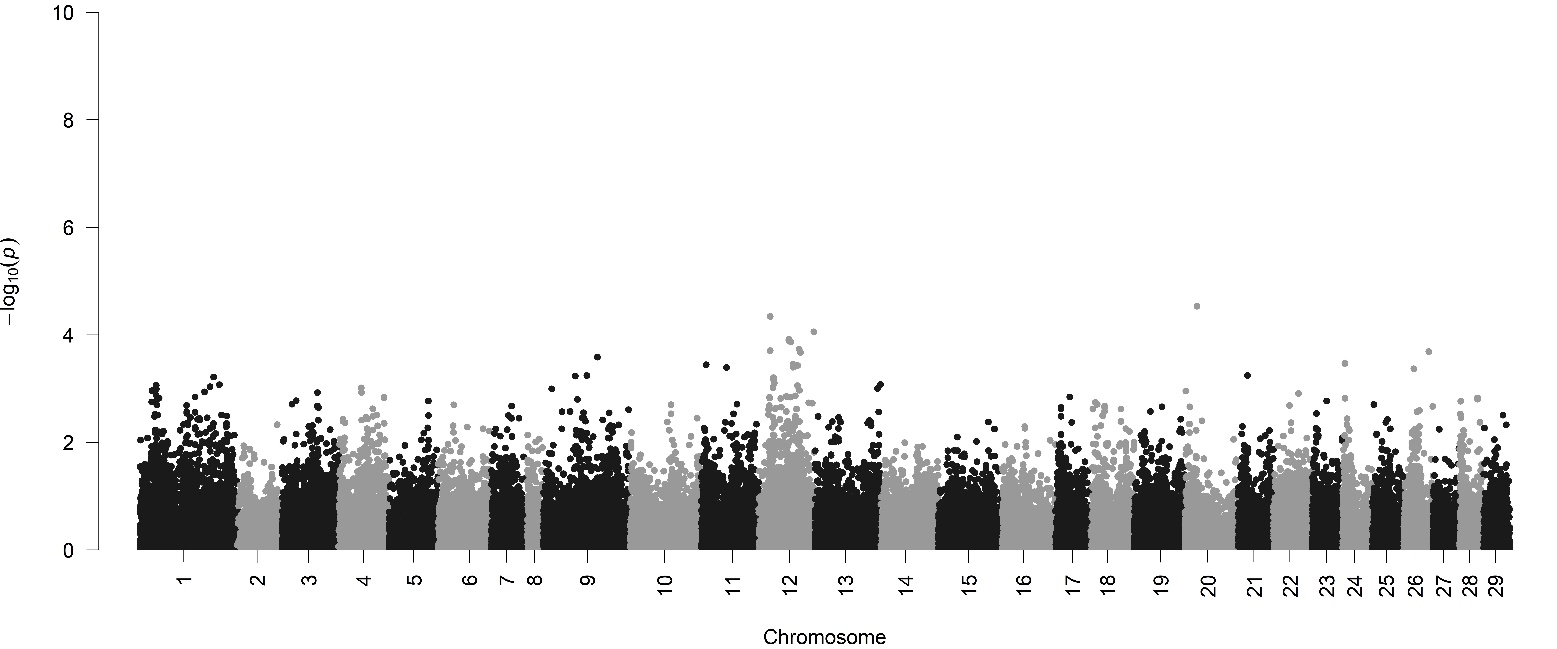


Figure S2: Manhattan plot of summary statistics derived from genome-wide association analysis for YC2016F


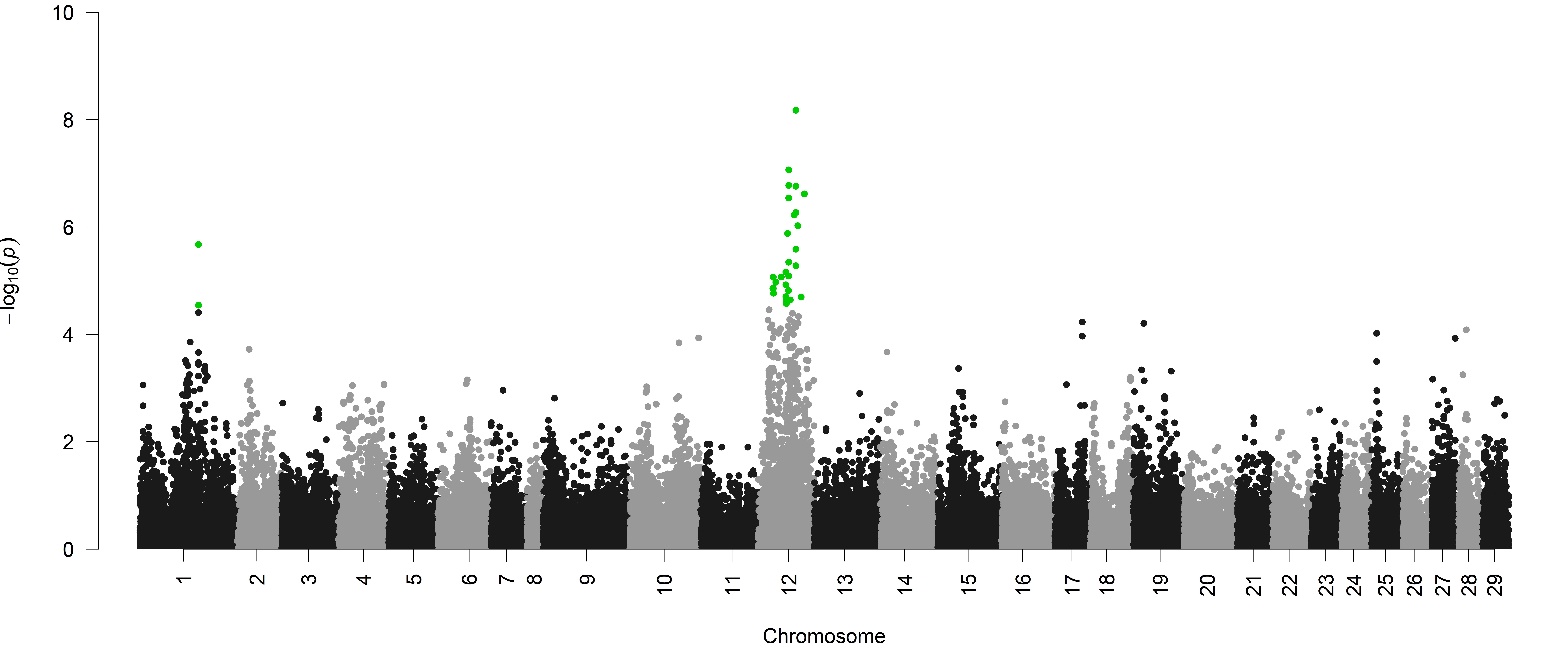


Figure S3: Manhattan plot of summary statistics derived from genome-wide association analysis for YC2017. Significant SNPs at FDR<0.05 are shown in green


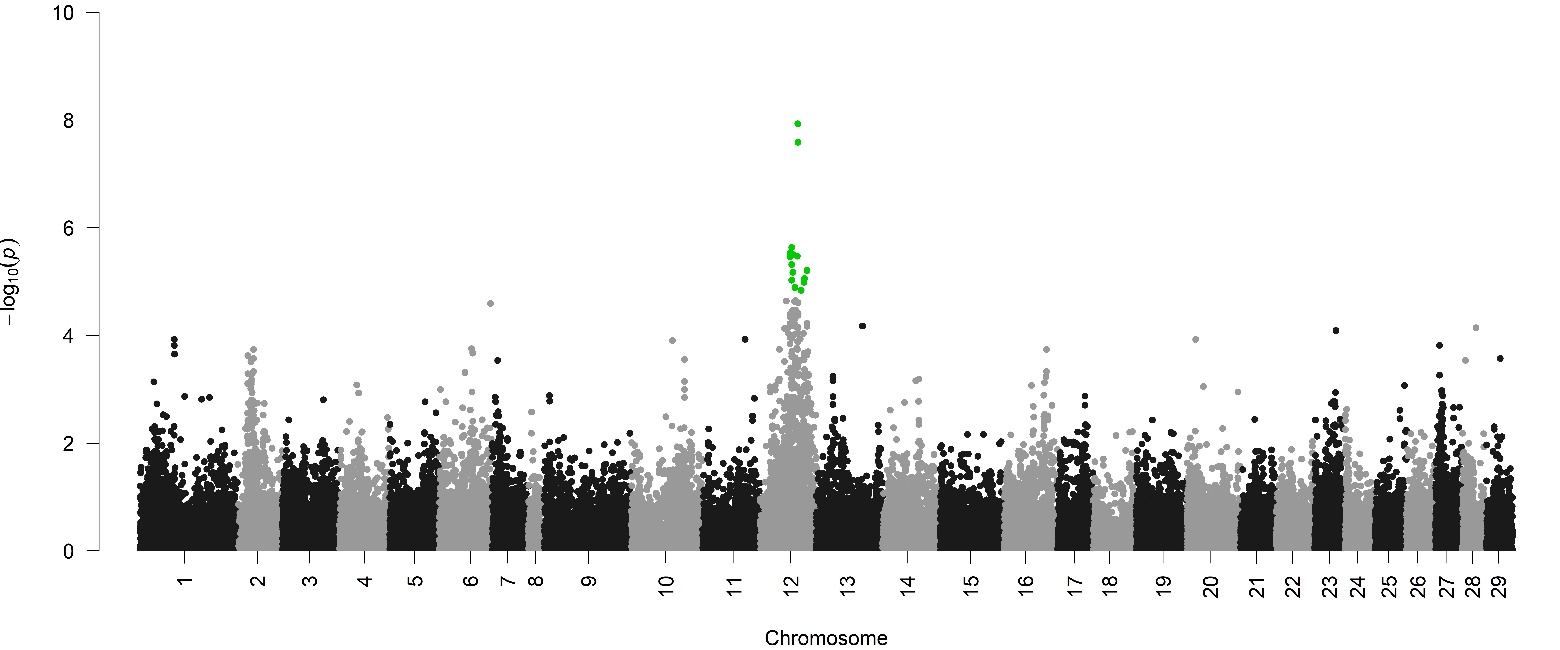


Figure S4: Manhattan plot of summary statistics derived from genome-wide association analysis for YC2018. Significant SNPs at FDR<0.05 are shown in green
