# Supplementary material for: Genome-wide association analysis using multiple Atlantic salmon populations
Source: Genet Sel Evol. 2025 Feb 27;57:9. doi: 10.1186/s12711-025-00959-1 (PMC11869457; doi:10.1186/s12711-025-00959-1)
Supplement: Supplementary file 3 — Additional file 3: Figure S5. Manhattan plot of summary statistics derived from mega genome-wide association analysis. Figure S6. Manhattan plot of summary statistics derived from Zscore meta-genome-wide association analysis. Figure S7. Manhattan plot of summary statistics derived from inverse invariance weighted meta-genome-wide association analysis. Figure S8. Manhattan plot of summary statistics derived from random effect meta-genome-wide association analysis. [file 12711_2025_959_MOESM3_ESM.docx]

**Genome-wide association analysis using multiple Atlantic salmon populations**

Afees A. Ajasa^1,2^, Hans M. Gjøen^2^, Solomon A. Boison^3^ and Marie Lillehammer^1^

^1^Nofima (Norwegian institute of Food, Fisheries and Aquaculture research), PO Box 210, N-1431 Ås, Norway

^2^Department of Animal and Aquacultural Sciences, Norwegian University of Life Sciences, 5003 NMBU, N-1432 Ås, Norway

^3^Mowi Genetics AS, Sandviksboder 77AB, Bergen, Norway


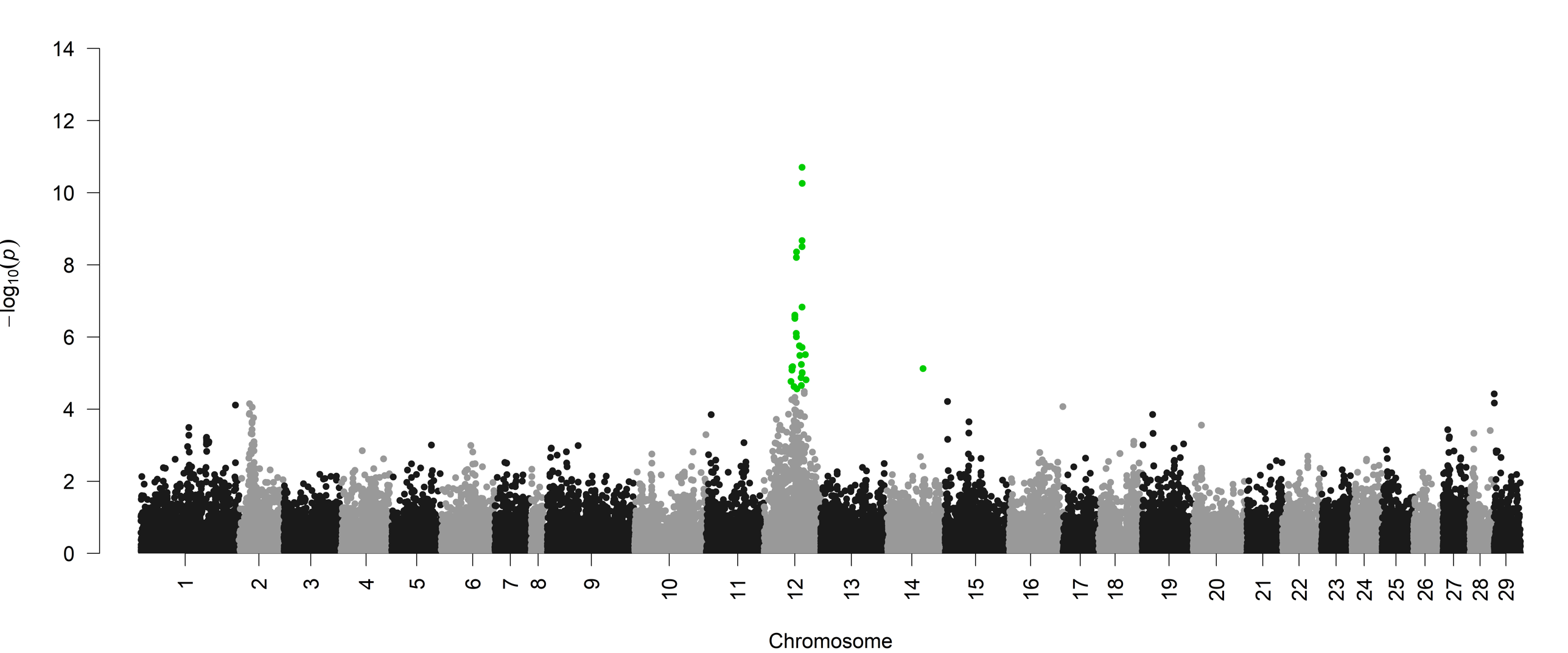


Figure S5: Manhattan plot of summary statistics derived from mega genome-wide association analysis


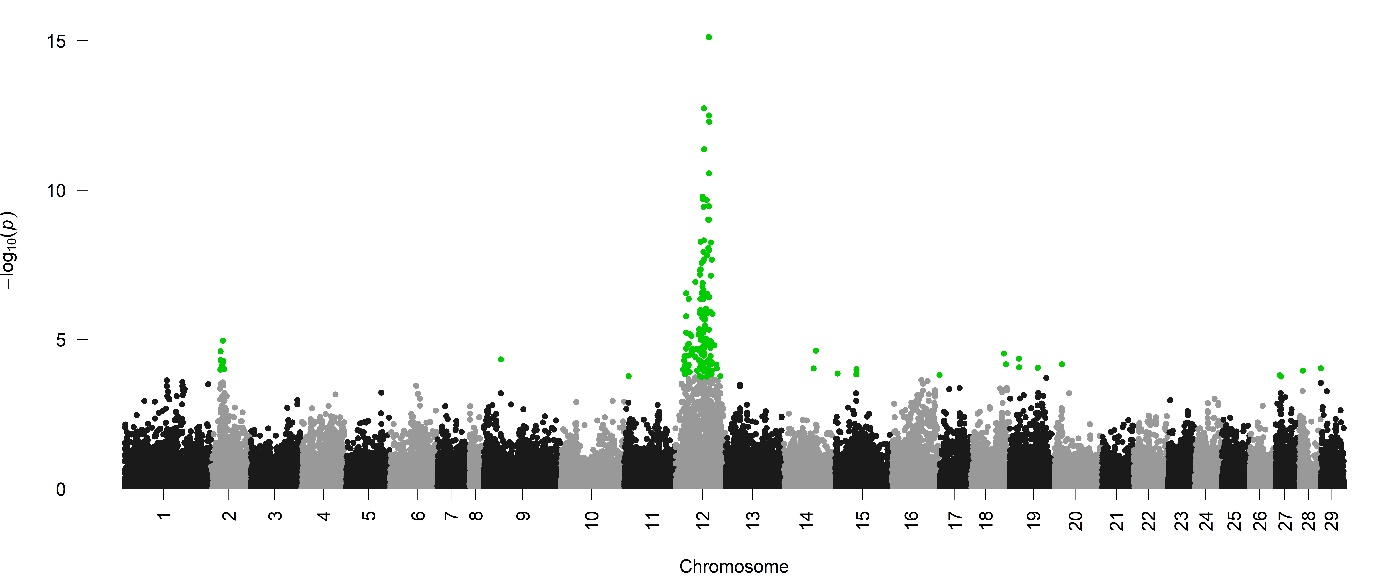


Figure S6: Manhattan plot of summary statistics derived from Zscore meta-genome-wide association analysis


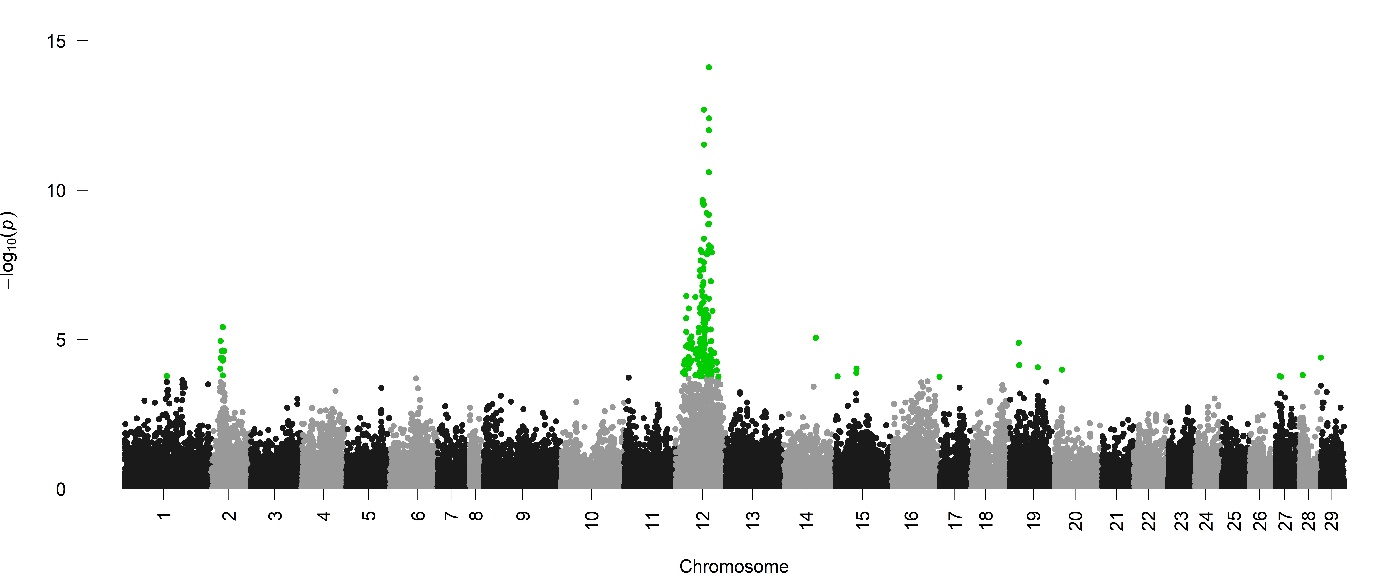


Figure S7: Manhattan plot of summary statistics derived from inverse invariance weighted meta-genome-wide association analysis


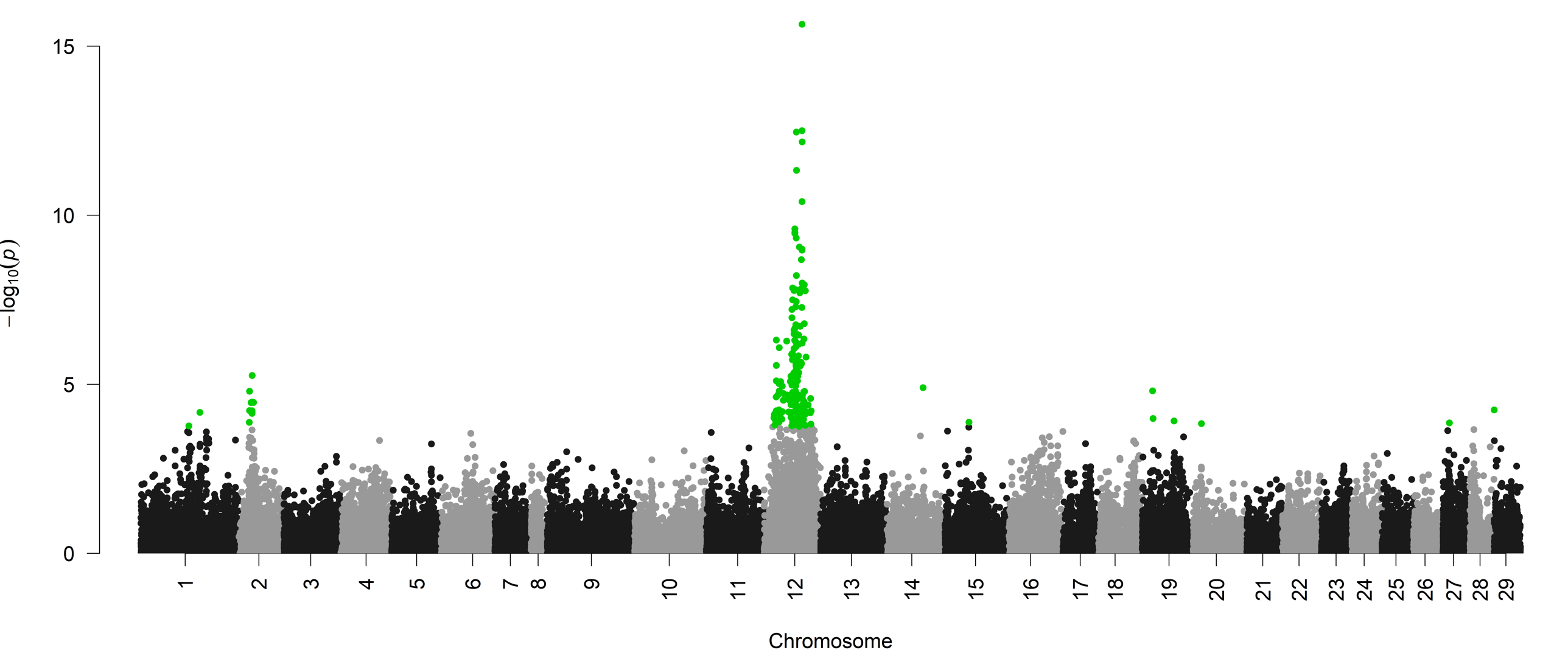


Figure S8: Manhattan plot of summary statistics derived from random effect meta-genome-wide association analysis
